# Clinical Significance of Color Ultrasound, MRI, miR-21, and CA199 in the Diagnosis of Pancreatic Cancer

**DOI:** 10.1155/2021/2380958

**Published:** 2021-07-28

**Authors:** Jing Yu, Xue Yang, Hongmei Wu, Jiansheng Li

**Affiliations:** Department of Abdominal Ultrasound, The Affiliated Hospital of Qingdao University, Qingdao 266000, China

## Abstract

**Background:**

To investigate the clinical significance of color ultrasound, magnetic resonance imaging (MRI), miR-21, and CA199 in the diagnosis of pancreatic cancer (PC).

**Methods:**

A total of 160 patients with PC admitted to our hospital from April 2018 to February 2021 were included in the PC group, and another 100 patients with benign pancreatic disease during the same period were included in the pancreatic benign disease group. Color ultrasound and MRI were used for imaging examination of the two groups of PC patients, and the sensitivity, accuracy, and specificity of the two methods for preoperative diagnosis of PC were calculated, respectively. A total of 100 healthy people who underwent physical examination during the same period were included in the control group. Serum CA199 levels of the three groups were detected by ELISA assay. The level of serum miR-21 in the three groups was detected by qRT-PCR. A receiver operating curve (ROC) was drawn to analyze and calculate the sensitivity, specificity, and accuracy of the two serum markers and the combination of color ultrasound and MRI in the detection of PC.

**Results:**

Serum CA199 and miR-21 levels in the PC group were significantly higher than those in the benign lesion group and control group. CA199 and miR-21 levels in the benign lesion group were higher than those in the control group. Both color ultrasound and MRI showed a higher detection rate for PC, and the sensitivity and accuracy were significantly higher than those of CA199 and miR-21. The sensitivity, specificity, and accuracy of combined detection were 91.88%, 96.00%, and 93.46%, respectively, which were significantly higher than those of single detection.

**Conclusion:**

The combined detection of color ultrasound, MRI, miR-21, and CA199 have a high application value in the early diagnosis of PC, which can effectively improve the sensitivity and accuracy of clinical diagnosis, reduce the probability of missed diagnosis and misdiagnosis, and provide a reference for the rational clinical treatment plan and prognosis.

## 1. Introduction

Pancreatic cancer (PC) is a malignant digestive tumor with high clinical morbidity and mortality and challenging to treat [1]. In recent years, due to changes in dietary habits, PC is more common in middle-aged and older adults, and the incidence has been increasing year by year [2]. The early clinical symptoms of PC have no apparent specificity, the clinical detection rate is low, and there are many cases of missed diagnosis and misdiagnosis. Most patients have been diagnosed in the middle and late stages. The particularity of the location of cancer makes patients lose the opportunity of radical surgery. Patients can only receive surgery and postoperative chemotherapy, with poor quality of life and poor prognosis [3]. Clinically, pathological tissue biopsy is used as the “gold standard” for the diagnosis of PC, but this examination method may cause a certain degree of trauma in patients and has limitations [4]. Therefore, early diagnosis and accurate assessment of preoperative staging of PC are crucial to the treatment effect and survival time of patients [5].

MiR-21 is abnormally expressed in a variety of malignant tumor tissues. MiR-21 plays an important role in the development and progression of PC, which can be used as a new target for diagnosing and treating PC [6]. At present, one of the methods commonly used in the clinical detection of various malignant tumors is the detection of serum tumor markers [7]. CEA [8] and CA199 [9] are widely used clinical serum markers for PC. However, CEA has a low sensitivity in the diagnosis of PC. Although CA199 has a high sensitivity in the diagnosis of PC, it has certain limitations [10]. Magnetic resonance imaging (MRI) is a common means for the clinical diagnosis of malignant tumors. With fast scanning speed and clear images, it can clearly show the situation of vascular invasion without ionizing radiation and can also be used for further observation of the surrounding tissues of the pancreas [11, 12]. Color ultrasound not only has the advantages of two-dimensional ultrasound structure image but also provides rich hemodynamic information, simple operation, and high accuracy of examination [13]. However, each method alone has advantages and limitations in the diagnosis of PC, and the combination of multiple methods can combine these advantages and significantly improve the diagnostic accuracy.

In this study, we studied the clinical diagnostic value of color ultrasound, MRI, miR-21, and CA199 combined examination in patients with PC, aiming to provide a reference for the early diagnosis, treatment, and prognosis of PC.

## 2. Materials and Methods

### 2.1. General Data

**Figure 1:**
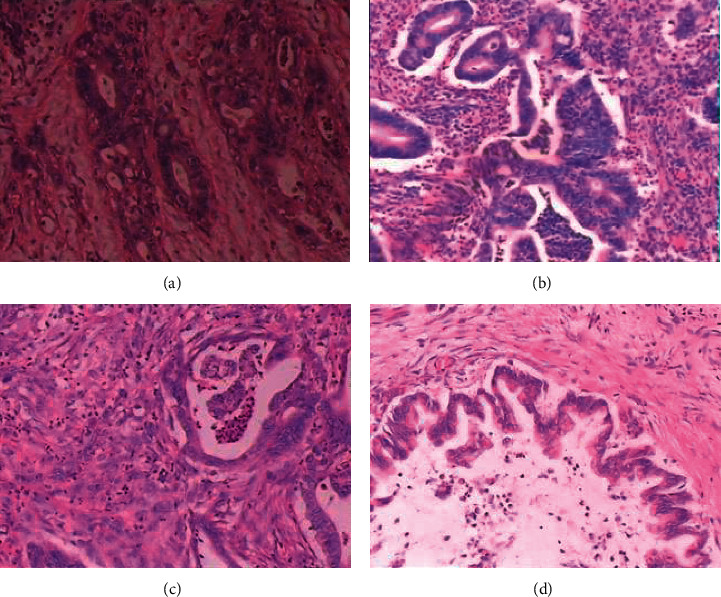
Pathologic biopsy of PC. (a) PC stage I in a 34-year-old woman with moderate to poorly differentiated adenocarcinoma (size 1.9 cm), not invading the pancreatic capsule. (b) PC stage II in a 57-year-old man with moderately differentiated adenocarcinoma (2.5 cm in diameter), invading the pancreatic capsule. (c) PC stage III in a 45-year-old male with moderately differentiated adenocarcinoma (31.5 cm) and high-grade ductal intraepithelial neoplasia extending into the pancreatic capsule to surrounding adipose tissue and the muscular layer. (d) PC stage IV in a 66-year-old male with stage IV pancreatic carcinoma presented with pathologic, moderately differentiated adenocarcinoma that invaded and punctured the pancreas.

A total of 160 patients with PC admitted to the Affiliated Hospital of Qingdao University (Qingdao, China) from April 2018 to February 2021 were included in the study group, aged from 26 to 71 years, with an average of 46.77 ± 10.65 years. The pathological biopsy results showed that 46 cases were TNM I stage, 68 cases were TNM II stage, 31 cases were TNM III stage, and 15 cases were TNM IV stage (Figure 1). Tumor diameter: 94 patients were <5 cm, and 66 patients were ≥5 cm. Types of disease: 131 cases were pancreatic head carcinoma, and 29 cases were pancreatic body carcinoma. Pathological types: 136 cases were ductal adenocarcinoma, 17 cases were mucinous adenocarcinoma, and 7 cases were adenosquamous carcinoma. Tumor differentiation: 45 cases were highly differentiated, 62 cases were moderately differentiated, and 53 cases were poorly differentiated. There were 83 patients with lymph node metastasis and 77 patients without lymph node metastasis. Vascular infiltration was found in 36 cases and not in 124 cases. Inclusion criteria: patients who did not receive surgical treatment before the examination; all patients confirmed by clinical examination and pathological examination; and complete clinical medical records. Exclusion criteria: patients with cirrhosis; patients with coagulation dysfunction or liver cancer metastatic; patients with cardiovascular and visceral diseases; complicated with other malignant tumors; confused consciousness or accompanied by mental illness; and patients with MRI contraindication.

A total of 100 patients with benign pancreatic lesions, aged from 25 to 74 years, with an average age of 47.52 ± 11.43 years, were included in the benign lesions group. Another 100 healthy subjects, aged from 26 to 73 years, with an average age of 48.13 ± 10.71 years, were enrolled into the control group during the same period. There was no statistical significance in the comparison of general information among the three groups (Table 1). All participants in this study signed the informed consent and were approved by the ethics committee of the Affiliated Hospital of Qingdao University (approval number: LIHAO:20180317).

### 2.2. CA199 and miR-21 Detection Methods

5 mL fasting venous blood in the morning was collected from the three groups and centrifuged at 3 000 r/min for 10 min. The upper serum was divided into two parts and stored in a refrigerator at −80°C for measurement. CA199 was detected by Enzyme Linked Immunosorbent Assay (ELISA). The ELISA kit was purchased from Sigma Company (United States), and the specific operation was strictly in accordance with the kit instructions.

MiR-21 level was detected by the qRT-PCR method. Total RNA was extracted by the Trizol method and dissolved in DEPC water, and its concentration and purity were detected by using an ultraviolet spectrophotometer. 1 *μ*g total RNA was used as a template to synthesize cDNA. Then, qRT-PCR was performed. The reaction conditions were as follows: 90°C for 2 min, 90°C for 20 s, 65°C for 30 s, 70°C for 10 s, and a total of 39 cycles. The primer sequences were miR-21 forward 5 ′-CGGCGGTTAGCTTATCAGACTGA-3′ and reverse 5 ′-CCAGTGCAGGGTCCGAGGGAGTAT-3′; U6 forward 5′-GCGCGTCGTG AAGCG TTC-3′ and reverse 5′- GTGCAGGGTCCGAGGT-3′. The results were analyzed by 2^−Δ^^Δ CT^.

### 2.3. Color Ultrasound Examination

The instrument for color ultrasound was Toshiba Aplio 500, and the probe frequency was set at 3.5 MHz. The patient was supine with the upper abdomen fully exposed and the entire epigastric area scanned. The internal diameters of intrahepatic and extrahepatic bile ducts and pancreatic ducts were measured, and space-occupying lesions in the ampulla and head of the pancreas were examined. The tumor location was determined, and the tumor size, shape, boundary, internal echo, lymph node metastasis, and its relationship with adjacent organs were observed. The size, contour, and shape of the pancreatic duct were examined. The subjects kept breathing deeply during the entire examination to obtain a clear image.

### 2.4. MRI Examination

The MRI instrument was a Ge3.0T Signa HDX MRI scanner. First, conventional *T*1 WI and *T*2 WI sequences were scanned. The scanning parameters of *T*1 WI were TR 295 ms, TE 3.5 ms, layer thickness 6 mm, layer spacing 3 mm, and matrix 256 × 256. The scanning parameters of *T*2 WI were TR 6000 ms, TE 90 ms, layer thickness 6 mm, layer spacing 3 mm, and matrix 325 × 226. The scanning range of both sequences was from the diaphragmatic apex to duodenal level. After the location of the lesion was determined, SE *T*1 WI and fat suppression sequence scanning were performed on the pancreatic layer. The thickness of the adjusted layer was 5 mm, and the spacing was 1 mm. Then dynamic enhanced scanning was performed, and the patient was injected with Magenweir contrast agent (1.5 mL/kg, 3 mL/s) through the cubital vein. The obtained imaging data will be uploaded to the workstation for image postprocessing. The final imaging data of all the subjects were reviewed by two experienced radiologists. For cases with inconsistent judgment results, the two doctors discussed or consulted experts and finally reached the same diagnosis results, and the final report was given.

### 2.5. Observation Indexes

The sensitivity, specificity, and accuracy of color ultrasound and MRI in the diagnosis of PC were compared, respectively. MiR-21 and CA199 levels in the PC group, benign lesion group, and control group were compared. The sensitivity, specificity, and accuracy of miR-21, CA199, combined with color ultrasound and MRI, in the detection of PC were compared. Sensitivity calculation method: true positive number/(true positive number + false negative number) × 100%. Specificity calculation method: true negative number/(true negative number + false positive number) × 100%. Accuracy calculation method: (true positive number + true negative number)/total number of cases × 100%.

### 2.6. Statistical Analysis

SPSS23.0 software was used for data analysis. Enumeration data were expressed as rate (%), and the *χ*2 test was used for comparison between groups. Measurement data were expressed as (*x* ± *s*), and the *χ*2 test was performed between groups. Receiver operating curve (ROC) was used to analyze the diagnostic value of miR-21 and CA199 in PC. *P* < 0.05 was considered statistically significant.

## 3. Results

### 3.1. Comparison of CA199 and miR-21 Levels in the Three Groups

**Figure 2:**
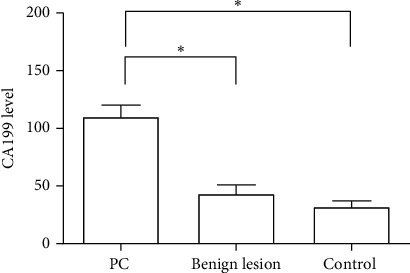
The level of CA199 in three groups.

**Figure 3:**
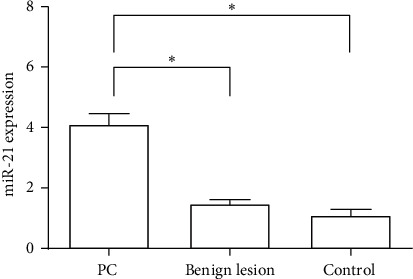
The expression of miR-21 in three groups.

Compared with the PC group, CA199 and miR-21 levels in the benign lesion group and control group were significantly downregulated (*P* < 0.01). CA199 and miR-21 levels were the lowest in the control group (Figures 2 and 3).

### 3.2. Color Ultrasound Images of PC Patients

**Figure 4:**
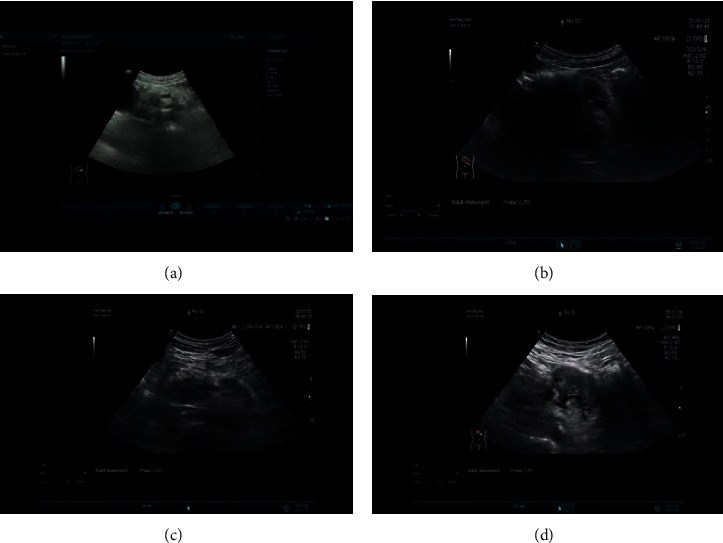
Color ultrasound image of a pancreatic cancer patient. (a) PC stage I: ultrasound showed hypoechoic nodules in the head of the pancreas with irregular shape and ill-defined boundaries, 1.9 × 1.7 cm. (b) PC stage II: ultrasound showed a hypoechoic nodule in the head of the pancreas with irregular shape and ill-defined boundaries, 2.6 × 1.7 cm. (c) PC stage III: ultrasound showed a low-echoing mass in the head and body of the pancreas, with irregular shape, poorly defined boundary, and poorly defined with surrounding tissues, 5.5 × 3.5 cm. (d) PC stage IV: ultrasound showed a hypoechoic mass in the tail of the pancreatic body, with irregular shape, ill-defined boundary, and unclear boundary with surrounding tissue, 6.9 × 4.2 cm, surrounded by adjacent arteries.

Color ultrasound images of patients with PC are as shown in Figure 4.

### 3.3. MRI Images of PC Patients

**Figure 5:**
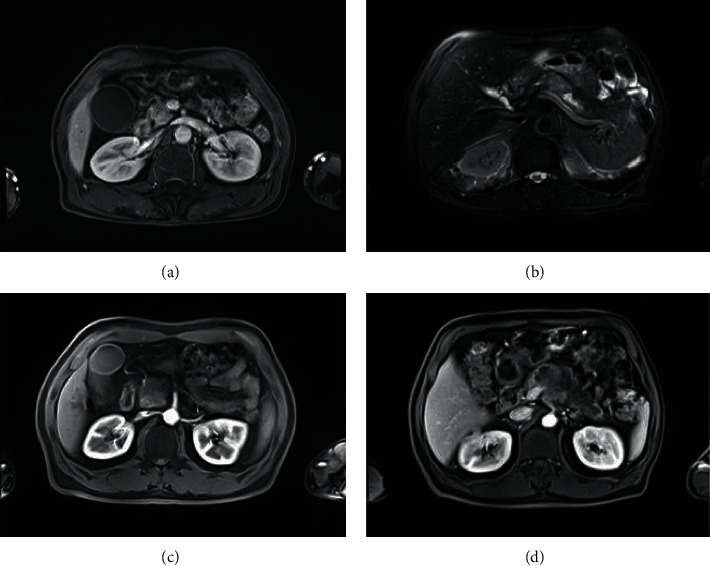
MRI images of patients with PC. (a) PC stage I: there is a nodular, slightly longer *T*1 shadow in the head of the pancreas, about 10 mm in diameter, with delayed enhancement and dilation of the main pancreatic duct. (b) PC stage II: there was a long *T*1 and *T*2 signal shadow in the body of the pancreas, with obvious hyperintensity on DWI and unclear boundary, 29 mm × 18 mm, and enhanced scanning showed mild enhancement and distal pancreatic duct dilation. (c) PC stage III: the pancreatic head is enlarged, and the pancreatic body and tail are atrophic. In the pancreatic head area, there are lump-like long *T*1 signal shadows, 43 mm × 40 mm, and mild enhancement. (d) PC stage IV: there were lump-like long *T*1 shadows in the body of the pancreas, with an unclear boundary, 40 mm × 85 mm, the enhancement degree was lower than that of the pancreatic parenchyma on an enhanced scan, and the adjacent peritoneal trunk was surrounded in it.

MRI images of patients with PC are as shown in Figure 5.

### 3.4. Comparison of the Diagnostic Value of Color Ultrasound and MRI in PC

Both color ultrasound and MRI had high detection rates for PC, and the difference between them was not statistically significant (*P* > 0.05, Tables 2 and 3).

### 3.5. Comparison of the Diagnostic Value of miR-21 and CA199 and Their Combined Detection in PC

**Figure 6:**
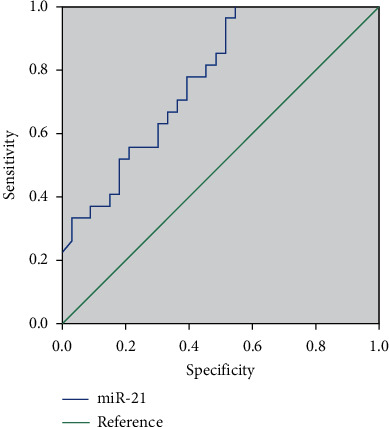
ROC curve of miR-21 in the diagnosis of PC.

**Figure 7:**
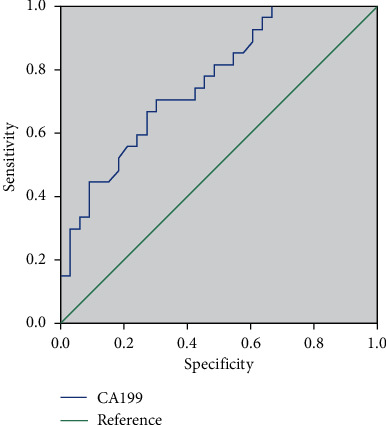
ROC curve of CA199 in the diagnosis of PC.

The sensitivity, specificity, and accuracy of miR-21 were 58.13%, 79.00%, and 66.15%, respectively (Figure 6). The sensitivity, specificity, and accuracy of CA199 were 69.38%, 74.00%, and 71.15%, respectively (Figure 7). The sensitivity, specificity, and accuracy of color ultrasonography were 83.13%, 89.00%, and 85.38%, respectively. The sensitivity, specificity, and accuracy of MRI were 81.25%, 90.00%, and 84.62%, respectively. The sensitivity, specificity, and accuracy of the combined detection were 91.88%, 96.00%, and 93.46%, respectively (Table 4). The sensitivity, specificity, and accuracy of the combined detection were all higher than those of the single detection.

## 4. Discussion

PC is one of the most common clinical malignant tumors of the digestive system. It has no typical features in the early stage and is highly occultive, which is easily ignored by patients [14]. With the extension of time, the disease began to deteriorate, and the patient had missed the best opportunity for radical treatment when diagnosed [15]. At present, the treatment of PC includes surgery, radiotherapy and chemotherapy, and targeted therapy. However, the treatment effect is poor, and the patients are prone to local recurrence and distant metastasis after surgery, and the 5-year survival rate is low, which seriously threatens the quality of human life [16]. The pancreas has a unique anatomical structure and biological characteristics, which are easily confused with other digestive system diseases. Currently, there is no effective and reliable method for diagnosing PC in clinical practice [17]. In recent years, with the continuous development of laboratory medicine and science and technology, some tumor markers can be applied in immunological detection, which increases the efficiency of early tumor diagnosis to a certain extent [18].

CA199 is currently the most commonly used marker for the diagnosis of pancreatic cancer. The expression level of CA199 is low in normal pancreatic tissues, but it is gradually upregulated when malignant lesions occur in pancreatic ductal epithelial cells [19]. With the continuous deterioration of the disease, the pancreatic duct and small pancreatic ducts are blocked by the tumor, and CA199 invades the stroma around the lesion, leading to a significant increase in the serum level of CA199 [20]. Ge et al. [21] showed that the serum CA199 level in the PC group was significantly higher than that in the pancreatic benign lesion group. Shi et al. [22] reported that serum CA199 levels in the PC group were significantly higher than those in the benign pancreatic disease group and normal control group. MiRNA is a class of short noncoding RNA molecules that can specifically recognize and bind to target genes and play a role in regulating the expression level of target genes [23]. Studies have reported that miR-21 is abnormally expressed in various malignant tumors, such as breast cancer and lung cancer, and can promote the proliferation, invasion, and metastasis of tumor cells [24, 25]. In this study, CA199 and miR-21 in the PC group were significantly upregulated compared with the benign disease group and control group, which was consistent with the abovementioned expression results. Our findings suggested that CA199 and miR-21 could be used as serological indicators for the clinical diagnosis of PC.

Ultrasound, MSCT, MRI, and other imaging techniques are widely used in the early diagnosis of PC. Imaging techniques can be used to directly observe the location, size, shape, invasion, and surrounding organ tissue of the lesion, so as to make an effective differential diagnosis and more accurate clinical staging evaluation of PC [26–28]. MRI is a chemical imaging technology, which has the advantages of high soft-tissue resolution, multiple imaging sequences and no ionizing radiation, etc. It has a better effect in detail display and can detect small lesions of PC, which has certain advantages in the diagnosis of PC [29]. Color ultrasound is a diagnostic technology based on two-dimensional ultrasound and has the characteristics of rapidness and noninvasiveness [30]. On color ultrasound, PC is characterized by smooth dilation and interruption of the pancreatic duct. PC is caused mainly by cancerous changes in the epithelial cells of the pancreatic duct. With the continuous deterioration of the cancer cells, the pancreatic duct in patients will be obstructed and narrowed, eventually resulting in smooth dilation and interruption of the pancreatic duct [31].

This study showed that the sensitivity, specificity, and accuracy of miR-21 were 58.13%, 79.00%, and 66.15%, respectively. The sensitivity, specificity, and accuracy of CA199 were 69.38%, 74.00%, and 71.15%, respectively. The sensitivity, specificity, and accuracy of color ultrasonography were 83.13%, 89.00%, and 85.38%, respectively. The sensitivity, specificity, and accuracy of MRI were 81.25%, 90.00%, and 84.62%, respectively. The sensitivity, specificity, and accuracy of the combined assay were 91.88%, 96.00%, and 93.46%, respectively. The detection rate of the combined assay was significantly higher than that of the single assay. The results showed that a single test in the diagnosis of PC has a higher rate of missed diagnosis and misdiagnosis, and the combination of multiple detection methods can make up for the limitations of each and significantly increase the sensitivity and accuracy of PC diagnosis. However, the sample size of this study is small, and it is a retrospective analysis, so there may be some deviation in the study results, and it is necessary to further improve the study in the future.

## 5. Conclusions

In conclusion, color ultrasound and MRI examination of PC have a high detection rate, providing imaging information for patients' treatment plans. Serum CA199 and miR-21 levels were significantly overexpressed in the serum of PC patients. Combined color ultrasound and MRI examination can significantly improve the sensitivity, specificity, and accuracy of the diagnosis of early PC, which is conducive to the clinical judgment of the severity of the disease of PC patients.

## Figures and Tables

**Table 1:** Comparison of general data of the three groups.

Group		PC group	Benign lesion group	Control group	*X* ^2^	*P* value
Cases		160	100	100		
Gender	Male	97	62	66	0.773	0.679
	Female	63	38	34		
Age (years)	≤45	76	57	55	2.654	0.265
	>45	84	43	45		
Education degree	Junior high school and below	62	31	34	1.718	0.424
	High school or above	98	69	66		
Occupation	Worker	107	68	71	1.917	0.751
	Self-employed	39	27	23		
	No job	14	5	6		

**Table 2:** Diagnosis of PC by color ultrasonography.

	Clinical stage				Total
	I stage	II stage	III stage	IV stage	
I stage	40	9	0	0	49
II stage	6	54	3	0	63
III stage	0	5	26	2	33
IV stage	0	0	2	13	15
Total	46	68	31	15	160

**Table 3:** Diagnosis of PC by MRI.

	Clinical stage				Total
	I stage	II stage	III stage	IV stage	
I stage	38	7	0	0	45
II stage	5	55	4	0	64
III stage	3	6	24	2	35
IV stage	0	0	3	13	15
Total	46	68	31	15	160

**Table 4:** Comparison of diagnostic value (%).

Diagnosis method	Sensitivity	Specificity	Accuracy	Positive prediction rate	Negative prediction rate
miR-21	58.13 (93/160)	79.00 (79/100)	66.15 (172/260)	81.58 (93/114)	54.11 (79/146)
CA199	69.38 (111/160)	74.00 (74/100)	71.15 (185/260)	81.02 (111/137)	60.16 (74/123)
Color ultrasonography	83.13 (133/160)	89.00 (89/100)	85.38 (222/260)	92.36 (133/144)	76.72 (89/116)
MRI	81.25 (130/160)	90.00 (90/100)	84.62 (220/260)	92.85 (130/140)	75.00 (90/120)
Combined diagnosis	91.88 (147/160)	96.00 (96/100)	93.46 (243/260)	97.35 (147/151)	88.07 (96/109)

## Data Availability

The data used to support the findings of this study are available from the corresponding author upon request.
